# Roles of Achieved Levels of Low-Density Lipoprotein Cholesterol and High-Sensitivity C-Reactive Protein on Cardiovascular Outcome in Statin Therapy

**DOI:** 10.1155/2019/3824823

**Published:** 2019-11-21

**Authors:** Myung Han Hyun, Yuchang Lee, Byoung Geol Choi, Jin Oh Na, Cheol Ung Choi, Jin Won Kim, Eung Ju Kim, Seung-Woon Rha, Chang Gyu Park, Eunmi Lee, Hong Seog Seo

**Affiliations:** ^1^Department of Internal Medicine, Korea University Guro Hospital, Seoul 152-703, Republic of Korea; ^2^Division of Cardiology, Department of Internal Medicine, Korea University Guro Hospital, Seoul 152-703, Republic of Korea; ^3^Division of Cardiology, Department of Internal Medicine, Wonkwang University Sanbon Hospital, Gyeonggi-do 435-040, Republic of Korea; ^4^Graduate School of Converging Science and Technology, Korea University–Korea Institute of Science and Technology (KU–KIST), Seoul 136-791, Republic of Korea; ^5^Future Convergence Research Division, Korea Institute of Science and Technology, Seoul 136-791, Republic of Korea

## Abstract

In statin therapy, the prognostic role of achieved low-density lipoprotein cholesterol (LDL-C) and high-sensitivity C-reactive protein (hsCRP) in cardiovascular outcomes has not been fully elucidated. A total of 4,803 percutaneous coronary intervention (PCI)-naïve patients who prescribed moderate intensity of statin therapy were followed up. Total and each component of major adverse cardiovascular events (MACE) according to LDL-C and hsCRP quartiles were compared. The incidence of 5-year total MACEs in the highest quartile group according to the followed-up hsCRP was higher than that in the lowest quartile (hazard ratio (HR) = 2.16, *p* < 0.001). However, there was no difference between the highest and lowest quartiles of the achieved LDL-C (HR = 0.95, *p* = 0.743). After adjustment of potential confounders, the incidence of total death, *de novo* PCI, atrial fibrillation, and heart failure in the highest quartile of followed-up hsCRP, was higher than that in the lowest quartile (all *p* < 0.05). However, other components except for *de novo* PCI in the highest quartile by achieved LDL-C was not different to that in the lowest quartile. These results suggest that followed-up hsCRP can be more useful for predicting future cardiovascular outcome than achieved LDL-C in PCI-naïve patients with statin therapy.

## 1. Introduction

Cardiovascular disease (CVD) is high in prevalence and is an important worldwide contributor to mortality and morbidity. The underlying lesion of atherosclerosis in CVD includes cholesterol and inflammatory cells, which levels in blood are related to clinical outcomes of CVD [1–3]. Epidemiological studies have shown that the association between low-density lipoprotein cholesterol (LDL-C) levels correlates to CVD risk [4]. Also, a genetic study using Mendelian randomization showed that serum LDL-C level has a significant impact on the clinical outcomes of CVD [5]. So far, it has been understood that cholesterol-lowering therapy including statin is a primary strategy in the prevention of CVD.

Recently, two major guidelines were announced for the prevention of CVD with cholesterol-lowering therapy, but these guidelines conflict regarding the use of the target levels for LDL-C in statin therapy [6, 7]. The European Society of Cardiology (ESC)/European Atherosclerosis Society (EAS) guideline suggested fixed target levels for the achieved LDL-C [6], adopting the result of a meta-analysis by the Cholesterol Treatment Trialists' Collaboration (CTTC) involving 170,000 patients [8], and recommends evaluating achieved LDL-C level to adjust statin intensity [9]. However, in the American College of Cardiology (ACC)/American Heart Association (AHA) guideline [7], there is no strategy for achieved LDL-C levels, due to lack of clinical trials that titrated statin intensity to specific LDL-C goals to improve CVD outcomes. Instead, the guideline emphasizes statin intensity according to the level of CVD risk to lower future CVD outcomes. Since many patients on statin therapy still have a high incidence of CVD outcomes, it seems that patients with high achieved LDL-C level have a high residual risk of CVD development [7]. In statin therapy, the range of LDL-C reduction is wide due to individual differences in balance between hepatic cholesterol synthesis and intestinal absorption [10–12]. However, only few studies reported the role of inter-individual responsiveness to statin therapy in CVD prevention with statin therapy.

On the other hand, high cholesterol *per se* in blood increases systemic inflammation. In response to hypercholesterolemia, the bone marrow and spleen increase production of inflammatory monocytes that enter the circulation, accumulate in lesions, and differentiate into macrophages [13–15]. The role of inflammation in driving the atherogenic response to hypercholesterolemia through the vulnerability of atherosclerotic plaque and progression to acute coronary syndrome has been clarified [16, 17]. Therefore, monitoring and control of inflammation is also important for the prevention of CVD. C-reactive protein (CRP) is a nonspecific inflammatory reactant produced by the liver. The increase in CRP is due to a rise in the plasma concentration of interleukin-6, produced predominantly by macrophages [18] and adipocytes [19]. At present, CRP is considered a risk marker for CVD in addition to being a prototypical marker of underlying inflammation [18]. However, the clinical significance of followed-up CRP level on future CVD risk in patients with ongoing statin therapy has not been fully determined.

Clinically, despite the fact that the underlying pathology of atherosclerosis in CVD is cholesterol retention and inflammation, the relationship between serum LDL-C level and CRP level is weak with and without statin therapy. In addition, although statins reduce both LDL-C and inflammation, majority of patients on statin therapy still have a higher achieved LDL-C than the target level, or CRP level than normal value, and whether these patients are still at high risk of CVD development is unknown. It is important to identify a proper surrogate marker of future CVD risk in patients with ongoing statin therapy. Therefore, in this study, we aimed to investigate whether the two candidate predictors, achieved LDL-C and high-sensitivity (hs) CRP, have different roles in predicting future CVD outcomes of percutaneous coronary intervention (PCI)-naïve patients who received statin therapy at the same intensity.

## 2. Results

### 2.1. Baseline Characteristics, Risk Factors, Laboratory Findings, and Co-Medications in the Study Population according to Quartiles of Achieved LDL-C and Followed-Up hsCRP Levels

The baseline characteristics of the 4,803 eligible patients across the quartiles of achieved LDL-C and followed-up hsCRP quartiles are described in Tables 1 and 2, Supplementary Tables [Supplementary-material supplementary-material-1] and [Supplementary-material supplementary-material-1]. According to LDL-C quartiles, the majority of the study population was at moderate-to-high risk of CVD, due to higher age (mean age, range 58.1–60.6 years), prevalence of diabetes (range 49.0–67.6%) and hypertension (range 64.2–70.6%) than in the general population. About 25% of the study subjects had angina pectoris, but had not received coronary intervention (Table 1). As shown in Table 2, the baseline clinical characteristics, risk factors, and comorbidities, statins with co-medication used, and laboratory findings in quartiles of the study population according to the followed-up hsCRP levels showed similar trend to those of the LDL-C. Overall, in the quartiles of the study population according to the achieved LDL-C levels, as achieved LDL-C increased, the proportion of male gender, age, presence of diabetes, use of antiplatelet agents, and ARB decreased, but with increasing followed-up hsCRP level, the proportion of male gender, age, presence of diabetes, triglyceride, and apolipoprotein B level, and medication with warfarin, diuretics, and ARB increased (Tables 1 and 2, Supplementary Tables [Supplementary-material supplementary-material-1] and [Supplementary-material supplementary-material-1]).

### 2.2. Comparison of Clinical Outcomes Associated with Achieved LDL-C and Followed-Up hsCRP Levels

**Figure 1:**
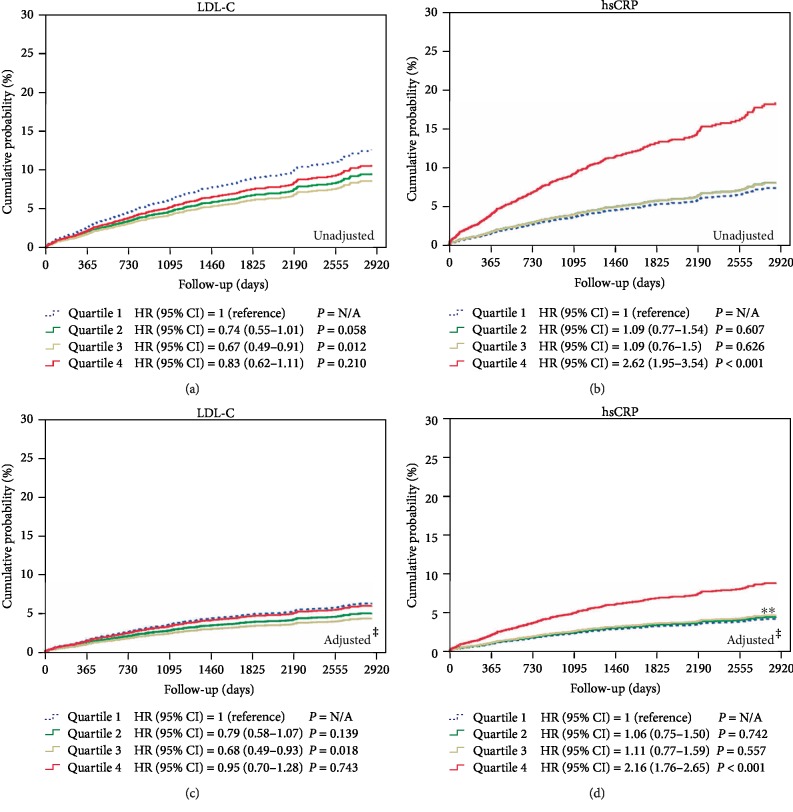
The cumulative incidences of total MACE, according to achieved LDL-C level and followed-up hsCRP level before and after adjustment of potential confounding factors. ^†^Major adverse cardiovascular event, was a composite of total death, acute myocardial infarction and cardiac death,* de novo *percutaneous coronary intervention, atrial fibrillation (AF), heart failure, and stroke. ^‡^Potential compounding factors are the following; age, sex, cardiovascular risk factors (HTN, DM, CKD, HF, AF, and angina pectoris), co-medications (aspirin, clopidogrel, cilostazol, warfarin, BB, diuretics, ARB, ACEI, CCB, nitrates, trimetazidine, nicorandil, and molsidomine). ^∗∗^Quartile 2 and Quartile 3 were slightly overlapped. MACE; major adverse cardiovascular events; LDL-C, low-density lipoprotein cholesterol; hsCRP, high-sensitivity C-reactive protein; HR, hazard ratio; CI, confidential intervals; N/A, not available; HTN, hypertension; DM, diabetes mellitus; CKD, chronic kidney disease; HF, heart failure; AF, atrial fibrillation; BB, beta blocker; ARB, angiotensin II receptor blocker; ACEi, angiotensin converting enzyme inhibitor; CCB, calcium channel.

The clinical follow-up duration was 1,750 ± 770 days. Before adjustment of the potential confounders, the 5-year incidence of MACEs in the highest quartile of the achieved LDL-C level was not higher than that in the lowest quartile of the achieved LDL-C level (hazard ratio (HR) = 0.83, 95% confidential intervals (CI) = 0.62–1.11, *p* = 0.210), but the 5-year incidence of MACEs in the highest quartile of the followed-up hsCRP level was higher than that in the lowest quartile of the followed-up hsCRP level (HR = 2.62, 95% CI = 1.95–3.54, *p* < 0.001), After adjustment of potential confounders such as age, sex (male), cardiovascular risk factors (hypertension, diabetes, chronic kidney disease, heart failure, AF, and angina pectoris), co-medications (aspirin, clopidogrel, cilostazol, warfarin, beta blockers, diuretics, ARB, ACEI, calcium channel blockers, nitrates, trimetazidine, nicorandil, and molsidomine), and calendar dates, the incidence of MACEs in the highest quartile group according to the followed-up hsCRP level was still higher than that in the lowest quartile of the followed-up hsCRP level (HR = 2.16, 95% CI = 1.76–2.65, *p* < 0.001), but not different between the highest and lowest quintiles of the achieved LDL-C level (HR = 0.95, 95% CI = 0.70–1.28, *p* = 0.743) (Figure 1).

**Figure 2:**
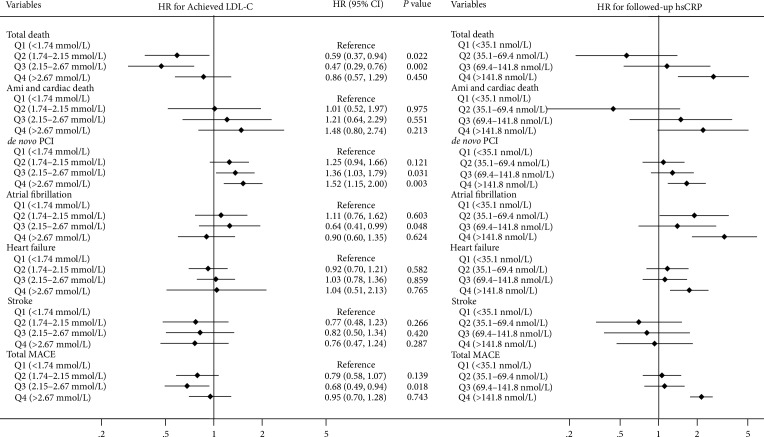
The 5-year incidence of total MACE and each component according to the achieved LDL-C and followed-up hsCRP. Subgroup analysis of the adjusted HR for the 5-year incidence of total death, AMI and cardiac death,* de novo *PCI, atrial fibrillation, heart failure, and stroke according to achieved LDL-C level and followed-up hsCRP level during the 5-year follow-up period, after moderate intensity of statin therapy. MACE; major adverse cardiovascular event, LDL-C; low density lipoprotein cholesterol, hsCRP; high-sensitivity C-reactive protein, HR; hazard ratio, CI; confidential intervals, Q; quartile, AMI; acute myocardial infarction, PCI; percutaneous coronary intervention.

The comparisons of 5-year incidence for each component of MACE (total death, myocardial infarction, sudden cardiac arrest, *de novo* PCI, new-onset persistent AF, new-onset heart failure, and stroke), to the quartiles according to achieved LDL-C levels and to the followed-up hsCRP levels are shown in Figure 2. After adjustment of the potential confounders, the 5-year incidence of total death, *de novo* PCI, AF, heart failure, in the highest quartile of the followed-up hsCRP levels were higher than those in the lowest quartile group (all *p* < 0.05). However, the 5-year incidence of only *de novo* PCI in the highest quartile groups according to the achieved LDL-C levels was higher than that in the lowest quartile group (*p* = 0.003). Therefore, achieved LDL-C level had a different association with CVD outcomes from that with followed-up hsCRP levels in patients with moderate statin therapy.

## 3. Discussion

In the present study, the followed-up hsCRP level was more widely associated with various CVD outcomes including death and myocardial infarction, *de novo* PCI, development of AF, and heart failure. On the other hands, achieved LDL-C was only associated with *de novo* PCI in patients with moderate statin use. Thus, followed-up hsCRP levels are more powerful predictors of future CVD risk than achieved LDL-C levels in PCI-naïve patients with ongoing moderate intensity statin therapy.

In addition to cholesterol lowering effect, statins improve endothelial function, increase vascular nitric oxide bioavailability, reduce oxidative stress, and improve endothelial progenitor cell function [20–22], and these nonlipid changing pleiotropic effects can also affect plaque vulnerability and CVD events. It is intriguing that the relationship between cholesterol level and CRP level remains very weak even with statin therapy, even with the fact that hypercholesterolemia induces systemic inflammation [13–15]. To date, numerous randomized controlled trials with statins and meta-analyses of trials using different intensities of statins assert that the amount of LDL-C lowering is related to CVD prevention, however, it is still uncertain that cholesterol and inflammation play different roles in clinical outcomes, because the amount of cholesterol lowering ranges widely among individuals at the same intensity of statin dose in clinical trials [10–12]. Ridker et al. showed that statin therapy results in a greater clinical benefit when levels of CRP are high, and that statins lower CRP levels in a manner largely independent of LDL-C levels [23]. In the PROVE IT-TIMI 22 (Pravastatin or Atorvastatin Evaluation and Infection Therapy-Thrombolysis In Myocardial Infarction 22) trial using either 80 mg of atorvastatin or 40 mg of pravastatin, the correlation between the achieved values was small, so that less than 3% of the variance in followed-up CRP levels was explained by the variance in achieved LDL-C levels [24]. However, the authors demonstrated a linear relationship between the levels of LDL-C achieved after statin therapy and the risk of CVD events, and a similar linear relationship between the levels of followed-up CRP and the risk of CVD, in spite of the almost complete independence of followed-up CRP and achieved LDL-C levels [24].

The exact mechanism of the result is not fully elucidated. It is reported that the response of cholesterol lowering to increases in the statin dose intensity is not proportional, because the dose-response relationship for all statins is curvilinear [25]. In general, a doubling above the minimal effective dose decreases serum LDL-C concentrations by an additional 6% [25]. This pharmacologic response may further potentiate inter-individual variability of achieved LDL-C levels with statin therapy. However, statins have dose-dependent pleiotropic effects [26, 27]. We postulate that this discrepancy between LDL-lowering and pleiotropic effects can explain the fact that the LDL-C change with statins is irrelevant to that of hsCRP. Therefore, when the effect of statins with achieved cholesterol level on CVD prevention is evaluated, it is reasonable to relate the achieved cholesterol level to the effect of CVD prevention differently according to statin intensity, and to adjust the dose-dependent pleiotropic effects of statins.

In the current study, nonetheless we showed that both LDL-C and hsCRP are associated with CVD outcomes, the associations are quite different. We evaluated the relationship of hsCRP level with the same statin intensity to CVD outcomes, and showed that the relationship with most CVD outcomes with achieved CRP level was persistent, whereas achieved LDL-C level was related only to *de novo* PCI procedures. In the JUPITER (Justification for the Use of Statins in Primary Prevention: An Intervention Trial Evaluating Rosuvastatin trial) trial, the authors stressed the importance of CRP level in future CVD events regardless of LDL-C level. In meta-analysis of clinical trials with various intensities of statins, the CTTC group concluded that every 1.0 mmol/L reduction in LDL-C is associated with a corresponding 22% reduction in atherosclerotic CVD mortality and morbidity [8]. However, we speculate that a different intensity of statin dose has an independent CVD reduction rate at the same level of achieved LDL-C, and that a considerable portion of statin therapy is overestimated or underestimated, depending upon statin intensity or LDL-C lowering. Therefore, future pooled analysis of statin trial needs to consider statin intensity to evaluate whether achieved LDL-C level is associated with a preventive effect in patients on statin therapy. In this context, the present study showed a powerful relationship between followed-up hsCRP level and various CVD outcomes compared with achieved LDL-C in ongoing statin therapy with moderate intensity.

It is interesting that the pleiotropic effect of statins is more predictive of clinical outcomes than the effect on cholesterol in the present study with moderate statin therapy. This result is associated with the definition of MACE as a composite with several clinical outcomes that may involve inflammation. Only *de novo* PCI, which is known to involve a pure atherosclerotic process, was dependent upon both achieved LDL-C and followed-up CRP levels; however, the contribution of de novo PCI was not so prominent in total MACE. Our data showed occurrence of death, myocardial infarction, AF, and heart failure are associated with inflammation, as well as *de novo* PCI procedures. Therefore, the majority of MACE is more dependent upon hsCRP levels. Thus, CRP level needed to be monitored during ongoing moderate statin therapy in patients with moderate-to-high risk.

In addition to inter-individual variation of LDL-C lowering and the pleiotropic effects of statin therapy, there is a substantial evidence that achieved LDL-C level is less reliable for prediction of future CVD events, considering the significance of intestine-derived cholesterol in CVD risk. Statins shift the cholesterol source from hepatic synthesis to intestinal absorption to buffer the effect of mevalonate inhibition [28]. Recent studies have shown that intestinal apolipoprotein-B48-containing chylomicron remnants contribute to atherogenesis and are a significant risk factor for CVD [29]. Several basic studies revealed the presence of intestinal cholesterol in atherosclerotic plaques [30–32], and clinical studies showed the significance of intestinal cholesterol in direct CVD risk [33–36]. A Mendelian randomized study also showed that intestine-derived cholesterol has an effect on CVD risk as strong as cholesterol from hepatic synthesis [37]. Intervention that reduced intestinal cholesterol also reduced CVD risks in clinical studies [38, 39]. Thus, increasing effects of intestinal cholesterol with statin therapy can offset the LDL-C lowering effect, and this can explain why LDL-C lowering is less relevant than CRP in prediction of CVD outcomes. One genetic study showed that LDL-C lowering by statins had only one-third of the CVD prevention effect of LDL-C lowering due to genetic effects [5]. Until now, CVD prevention effects with statin therapy were considered to be predominantly due to LDL-C lowering, and the pleiotropic effect of statins was considered to be a secondary phenomenon related to LDL-C lowering. Therefore, we assume that it is difficult to separate these statin effects. However, the results of the present study suggest that the CVD prevention effect of statins is more dependent upon pleiotropic effects than LDL-C lowering.

This study also has some limitations. It is based on single center, observational registry data. However, we carefully select a study population with the same intensity of statin therapy and adjust various potential confounders to overcome a weakness of previous trials. Nonetheless, this study was designed to generate a hypothesis to clarify the role of achieved LDL-C level and followed-up hsCRP level using different intensities of statin dose. Secondly, as shown in our data, the effect of CRP on CVD events was consistently high, when followed-up hsCRP was higher than 2 mg/L, regardless of LDL-C level in ongoing statin therapy. Further increments of statin intensity can reduce CVD events. It is also important to note that the study subjects were at moderate-to-high risk, with older age at around 60, and with a high prevalence of diabetes and hypertension, all of which are well known to be associated with chronic inflammation independent of cholesterol level, despite the adjustment of compounding factors. Therefore, the results of this study are not applicable to all age groups, and further studies are needed. Lastly, although we enrolled only Asian population, the data is not sufficient to apply the result to other race. Despite these limitations, the results of this study are important for the prevention of CVD, because the role of statins in preventing clinical outcomes was identified, independent of LDL-C level achieved with statin therapy; therefore, this result can relieve some concerns about whether we should monitor the cholesterol and hsCRP levels with statin use in clinical practice.

In conclusion, statins reduced LDL-C level, hsCRP level, and cardiovascular clinical outcomes. However, the effect of achieved LDL-C levels on CVD outcomes is less than that for followed-up hsCRP level in patients with ongoing statin therapy. Therefore, hsCRP level would be recommended as a surrogate predictive marker for future CVD events of PCI-naïve patients with statin therapy.

## 4. Materials and Methods

### 4.1. Study Population, Definition of Risk Factors, and Clinical Follow-Up

The study protocol conforms to the ethical guidelines of the Declaration of Helsinki and its later amendments or comparable ethical standards. The study protocol has been approved by the institutional review board of ethics committee on research on humans of Korea University Guro Hospital (#KUGH15312).

Between January 2005 and February 2014, a consecutive 29,175 statin-naive patients visited the Cardiovascular Center at the Korea University Guro Hospital in Seoul, South Korea. Among, 13,503 received moderate-intensity statin treatment (atorvastatin, simvastatin, or rosuvastatin) for at least 6 months after baseline lipid profile testing and were followed up clinically for at least 5 years. All patients in the cohort were followed up in the outpatient department of Korea University Guro Hospital. Among the patients, 1,023 who underwent a previous PCI were excluded. In addition, 968 patients who did not have data on their achieved LDL-C level and 7,267 patients who did not measure followed-up hsCRP level were excluded (558 patients missed both measurements). Finally, a total of 4,803 patients, who had not received PCI but prescribed moderate intensity of statin therapy with available data on both baseline blood lipids and hsCRP values, were finally enrolled (Supplementary [Supplementary-material supplementary-material-1]). Demographic data and risk factors such as the presence of a previous myocardial infarction, coronary spasm, heart failure, peripheral arterial disease, chronic kidney disease, and stroke, as well as medications, were also evaluated. Hypertension was defined as a systolic blood pressure of ≥140 mm Hg and/or a diastolic blood pressure of ≥90 mm Hg on at least two consecutive readings in the outpatient clinic. Diabetes was defined as a fasting blood glucose level ≥126 mg/dL, a glycated hemoglobin A1c level >6.5%, or current use of medications. The serum lipid profile, including LDL-C, high-density lipoprotein cholesterol, and triglyceride, fasting glucose, and serum hsCRP levels were measured by using chemiluminescence (Immulite; DPC Cirrus Inc., Los Angeles, CA, USA). High-, moderate-, and low-intensity statin therapies were defined according to the ACC/AHA guidelines on the treatment of blood cholesterol at any time during the study [7]. As previous studies showed that Asians may not need a higher intensity of statin therapy than that given to Caucasians to achieve target LDL-C levels owing to their different genetic and clinical backgrounds, most Asians received a moderate- instead of high-intensity statin therapy in real clinical practice [40–42]. Therefore, we exclusively included patients who received moderate-intensity of statins.

The achieved LDL-C and followed-up hsCRP values taken at baseline and after 6 to 9 months of treatment with statin therapy were recorded. The demographic data, cardiovascular risk factors, and medical history records were mainly dependent on patient self-reporting, but the final records were left to physician discretion after all of the subjects had comprehensive evaluation of self-reported data and in-hospital examination results. The primary endpoint was a total major adverse cardiovascular event (MACE), i.e., the incidence of cumulative clinical events such as composite of total death, myocardial infarction, sudden cardiac arrest, *de novo* PCI, new-onset persistent atrial fibrillation (AF), new-onset heart failure, and stroke, and the secondary endpoint was the incidence of each clinical event of the primary endpoint.

### 4.2. Statistical Analysis for the Laboratory and Clinical Data

For continuous variables, differences between two groups were evaluated by using Student's *t-*test or the Mann-Whitney rank sum test, and those between three groups were evaluated by using the one-way analysis of variance. Data were expressed as mean ± standard deviation. For discrete variables, differences were expressed as counts and percentages and analyzed by using either the chi-square or Fisher's exact test, when appropriate. In order to adjust for potential confounders, multivariate Cox proportional regression analysis was performed. We tested all available variables that could be of potential relevance: age, sex (male), cardiovascular risk factors (hypertension, diabetes, chronic kidney disease, heart failure, AF and angina pectoris), co-medications (aspirin, clopidogrel, cilostazol, warfarin, beta blockers, diuretics, angiotensin receptor blockers (ARB), angiotensin-converting-enzyme inhibitors (ACEI), calcium channel blockers, nitrates, trimetazidine, nicorandil, and molsidomine), and calendar dates. The incidence of total MACEs and each component at 5-year follow-up was estimated by using the Kaplan-Meier method, and between-group differences were compared using the log-rank test. For the adjustment of compounding factors, the total MACEs were assessed using multivariate Cox proportional hazard regression models. All statistical analyses were performed using SPSS 20.0 statistical software (SPSS Inc., Chicago, IL, USA).

## Figures and Tables

**Table 1:** Baseline characteristics and statins usage according to quartiles of achieved LDL-C level.

	LDL-C				
Variables	Quartile 1 (<1.74 mmol/L)	Quartile 2 (1.74–2.15 mmol/L)	Quartile 3 (2.15–2.67 mmol/L)	Quartile 4 (>2.67 mmol/L)	*p* value
Male, *n* (%)	701 (57.2)	593 (49.6)	542 (45.7)	509 (42.6)	<0.001
Age, year	60.6 ± 10.7	59.9 ± 10.6	59.5 ± 10.9	58.1 ± 11.2	<0.001
BMI, kg/m^2^	24.3 ± 3.4	24.6 ± 3.3	24.7 ± 3.2	24.7 ± 3.4	0.285
HTN, *n* (%)	854 (69.7)	845 (70.7)	862 (72.6)	767 (64.2)	0.017
DM, *n* (%)	829 (67.6)	624 (52.2)	582 (49.0)	602 (50.4)	<0.001
Insulin	262 (21.4)	178 (14.9)	166 (14.0)	176 (14.7)	<0.001
Oral medication	769 (62.7)	564 (47.2)	499 (42.0)	515 (43.1)	<0.001
Newly diagnosed	40 (3.3)	49 (4.1)	66 (5.6)	69 (5.8)	0.001
CKD, *n* (%)	116 (9.5)	114 (9.5)	106 (8.9)	111 (9.3)	0.770
eGFR, mL/min	92.2 ± 30.1	92.4 ± 29.1	90.4 ± 27.3	89.9 ± 27.1	0.102
HF, *n* (%)	73 (6.0)	56 (4.7)	52 (4.4)	51 (4.3)	0.051
Persistent AF, *n* (%)	41 (3.3)	47 (3.9)	44 (3.7)	43 (3.6)	0.815
Angina pectoris, *n* (%)	342 (27.9)	353 (29.5)	320 (27.0)	277 (23.2)	0.004
Chest pain, *n* (%)	302 (24.6)	303 (25.3)	279 (23.5)	235 (19.7)	0.002
Vasospastic angina, *n* (%)	40 (3.3)	50 (4.2)	41 (3.5)	42 (3.5)	0.978

*Laboratory findings*					
TC, mmol/L	3.13 ± 0.47	3.65 ± 0.36	4.14 ± 0.36	5.21 ± 0.75	<0.001
TG, mmol/L	1.36 ± 1.02	1.39 ± 0.86	1.56 ± 0.95	1.83 ± 0.97	<0.001
HDL-C, mmol/L	1.30 ± 0.36	1.32 ± 0.31	1.35 ± 0.31	1.37 ± 0.34	<0.001
LDL-C, mmol/L	1.42 ± 0.26	1.94 ± 0.13	2.41 ± 0.16	3.37 ± 0.62	<0.001
hsCRP, nmol/L	31.43 ± 95.24	23.81 ± 79.05	24.76 ± 106.67	28.57 ± 89.53	0.156
Fasting glucose, mmol/L	6.88 ± 2.05	6.66 ± 2.05	6.66 ± 2.11	6.83 ± 2.61	0.018
HbA1c, %	6.9 ± 1.2	6.7 ± 1.2	6.7 ± 1.3	6.8 ± 1.3	0.002
Cr, *μ*mol/L	76.3 ± 83.9	76.3 ± 91.5	76.3 ± 91.5	76.3 ± 83.9	0.949
Lipoprotein (a), *μ*mmol/L	0.71 ± 0.71	0.93 ± 1.00	1.07 ± 1.07	1.25 ± 1.36	<0.001
Apo A-I, g/L	1.34 ± 0.28	1.41 ± 0.25	1.45 ± 0.22	1.49 ± 0.26	<0.001
Apo B, g/L	0.50 ± 0.10	0.62 ± 0.09	0.74 ± 0.10	0.95 ± 0.19	<0.001
Apo C-II, g/L	0.03 ± 0.01	0.04 ± 0.02	0.04 ± 0.02	0.05 ± 0.02	<0.001
Apo E, g/L	0.02 ± 0.018	0.03 ± 0.01	0.03 ± 0.01	0.04 ± 0.01	<0.001

*Type of statins*					<0.001
Atorvastatin, *n* (%)	617 (50.3)	650 (54.3)	601 (50.6)	535 (44.8)	
Simvastatin, *n* (%)	251 (20.5)	243 (20.3)	243 (20.5)	274 (22.9)	
Rosuvastatin, *n* (%)	243 (19.8)	175 (14.6)	145 (12.2)	191 (16.0)	
Pitavastatin, *n* (%)	60 (4.9)	70 (5.9%)	103 (8.7%)	88 (7.4)	
Pravastatin, *n* (%)	25 (2.0)	34 (2.8)	51 (4.3)	67 (5.6)	
Fluvastatin, *n* (%)	30 (2.4)	24 (2.0)	44 (3.7)	39 (3.3)	
Follow-up, days	1,639 ± 751	1,682 ± 769	1,792 ± 755	1,887 ± 803	<0.001

LDL-C, low-density lipoprotein cholesterol; BMI, body mass index; HTN, hypertension; DM, diabetes mellitus; CKD, chronic kidney disease; eGFR, estimated glomerular filtration rate; HF, heart failure; AF, atrial fibrillation; TC, total cholesterol; TG, triglyceride; HDL-C, high-density lipoprotein cholesterol; hsCRP, high-sensitivity C-reactive protein; HbA1c, glycated hemoglobin; Cr, creatinine; Apo, apolipoprotein.

**Table 2:** Baseline characteristics and statins usage according to quartiles of followed-up hsCRP level.

	hsCRP				
Variables	Quartile 1 (<35.1 nmol/L)	Quartile 2 (35.1–69.4 nmol/L)	Quartile 3 (69.4–141.8 nmol/L)	Quartile 4 (>141.8 nmol/dL)	*p* value
Male, *n* (%)	543 (44.4)	570 (47.9)	625 (52.3)	607 (50.8)	<0.001
Age, year	59.3 ± 10.5	59.9 ± 10.5	58.7 ± 10.5	60.3 ± 11.9	0.001
BMI, kg/m^2^	24.3 ± 3.0	24.6 ± 3.2	24.7 ± 3.3	24.9 ± 3.7	0.059
HTN, *n* (%)	877 (71.7)	821 (69.0)	770 (64.4)	860 (72.0)	0.535
DM, *n* (%)	460 (37.6)	622 (52.3)	799 (66.8)	756 (63.3)	<0.001
Insulin	103 (8.4)	160 (13.5)	243 (20.3)	276 (23.1)	<0.001
Oral medication	406 (33.2)	543 (45.7)	731 (61.1)	667 (55.9)	<0.001
Newly diagnosed	49 (4.0)	60 (5.0)	52 (4.3)	63 (5.3)	0.248
CKD, *n* (%)	92 (7.5)	95 (8.0)	85 (7.1)	175 (14.7)	<0.001
eGFR, mL/min	91.7 ± 25.6	90.9 ± 26.1	94.7 ± 26.7	87.9 ± 34.0	<0.001
HF, *n* (%)	54 (4.4)	48 (4.0)	40 (3.3)	90 (7.5)	0.002
Persistent AF, *n* (%)	49 (4.0)	31 (2.6)	30 (2.5)	65 (5.4)	0.086
Angina pectoris, *n* (%)	408 (33.3)	301 (25.3)	265 (22.2)	318 (26.6)	<0.001
Chest pain, *n* (%)	345 (28.2)	261 (22.0)	227 (19.0)	286 (24.0)	0.004
Vasospastic angina, *n* (%)	63 (5.1%)	40 (3.4)	38 (3.2)	32 (2.7)	0.001

*Laboratory findings*					
TC, mmol/L	3.96 ± 0.85	4.04 ± 0.88	3.99 ± 0.91	4.07 ± 1.04	0.073
TG, mmol/L	1.28 ± 0.70	1.51 ± 0.93	1.63 ± 1.12	1.68 ± 1.06	<0.001
HDL-C, mmol/L	1.40 ± 0.34	1.35 ± 0.34	1.30 ± 0.31	1.27 ± 0.34	<0.001
LDL-C, mmol/L	2.25 ± 0.73	2.28 ± 0.78	2.25 ± 0.80	2.33 ± 0.85	0.011
hsCRP, nmol/L	2.7 ± 1.0	6.7 ± 1.0	12.4 ± 2.7	86.7 ± 173.3	<0.001
Fasting glucose, mmol/L	6.27 ± 1.78	6.66 ± 1.94	7.10 ± 2.28	7.05 ± 2.72	<0.001
HbA1c, %	6.5 ± 1.1	6.7 ± 1.1	6.9 ± 1.3	7.0 ± 1.4	<0.001
Cr, *μ*mol/L	68.6 ± 76.3	68.6 ± 68.6	76.3 ± 83.9	91.5 ± 114.4	<0.001
Lp (a), *μ*mmol/L	1.04 ± 1.18	0.93 ± 0.89	0.96 ± 1.07	1.11 ± 1.21	0.248
Apo A-I, g/L	1.46 ± 0.23	1.44 ± 0.25	1.40 ± 0.26	1.39 ± 0.28	0.013
Apo B, g/L	0.67 ± 0.20	0.67 ± 0.20	0.70 ± 0.21	0.73 ± 0.23	0.002
Apo C-II, g/L	0.04 ± 0.02	0.04 ± 0.02	0.04 ± 0.02	0.04 ± 0.02	0.275
Apo E, g/L	0.03 ± 0.01	0.03 ± 0.01	0.03 ± 0.01	0.03 ± 0.01	0.016

*Type of statins*					0.009
Atorvastatin, *n* (%)	628 (51.3)	573 (48.2)	644 (53.8)	558 (46.7)	
Simvastatin, *n* (%)	264 (21.6)	265 (22.3)	229 (19.1)	253 (21.2)	
Rosuvastatin, *n* (%)	162 (13.2)	187 (15.7)	178 (14.9)	227 (19)	
Pitavastatin, *n* (%)	91 (7.4)	81 (6.8)	77 (6.4)	72 (6.0)	
Pravastatin, *n* (%)	48 (3.9)	50 (4.2)	33 (2.8)	46 (3.9)	
Fluvastatin, *n* (%)	31 (2.5)	33 (2.8)	35 (2.9)	38 (3.2)	
Follow-up, days	1,817 ± 736	1,865 ± 788	1,605 ± 760	1,709 ± 791	<0.001

LDL-C, low-density lipoprotein cholesterol; BMI, body mass index; HTN, hypertension; DM, diabetes mellitus; CKD, chronic kidney disease; eGFR, estimated glomerular filtration rate; HF, heart failure; AF, atrial fibrillation; TC, total cholesterol; TG, triglyceride; HDL-C, high-density lipoprotein cholesterol; hsCRP, high-sensitivity C-reactive protein; HbA1c, glycated hemoglobin; Cr, creatinine; Lp, lipoprotein; Apo, apolipoprotein.

## Data Availability

The data used to support the findings of this study are available from the corresponding author upon request.

## Supplementary Materials

Supplementary Materials Table S1: baseline characteristics of co-medications usage according to quartiles of achieved LDL-C level. Table S2: baseline characteristics of co-medications usage according to quartiles of followed-up hsCRP level. Figure S1: study flow chart.

## References

[1] Stamler J., Wentworth D., Neaton J. D. (1986). Is relationship between serum cholesterol and risk of premature death from coronary heart disease continuous and graded? Findings in 356,222 primary screenees of the Multiple Risk Factor Intervention Trial (MRFIT). *JAMA: The Journal of the American Medical Association*.

[2] Castelli W. P. (1984). Epidemiology of coronary heart disease: the Framingham study. *The American Journal of Medicine*.

[3] Verschuren W. M., Jacobs D. R., Bloemberg B. P. (1995). Serum total cholesterol and long-term coronary heart disease mortality in different cultures. Twenty-five-year follow-up of the seven countries study. *JAMA: The Journal of the American Medical Association*.

[4] Grundy S. M., Cleeman J. I., Merz C. N. (2004). Implications of recent clinical trials for the National Cholesterol Education Program Adult Treatment Panel III guidelines. *Circulation*.

[5] Ference B. A., Yoo W., Alesh I. (2012). Effect of long-term exposure to lower low-density lipoprotein cholesterol beginning early in life on the risk of coronary heart disease: a Mendelian randomization analysis. *Journal of the American College of Cardiology*.

[6] Catapano A. L., Graham I., De Backer G. (2016). 2016 ESC/EAS guidelines for the management of dyslipidaemias. *European Heart Journal*.

[7] Grundy S. M., Stone N. J., Bailey A. L. (2019). 2018 AHA/ACC/AACVPR/AAPA/ABC/ACPM/ADA/AGS/APhA/ASPC/NLA/PCNA guideline on the management of blood cholesterol: a report of the American College of Cardiology/American Heart Association Task Force on Clinical Practice Guidelines. *Circulation*.

[8] Treatment Cholesterol Trialists’ (CTT) Collaboration, Baigent C., Blackwell L. (2010). Efficacy and safety of more intensive lowering of LDL cholesterol: a meta-analysis of data from 170,000 participants in 26 randomised trials. *Lancet*.

[9] Navarese E. P., Robinson J. G., Kowalewski M. (2018). Association between baseline LDL-C level and total and cardiovascular mortality after LDL-C lowering: a systematic review and meta-analysis. *The Journal of the American Medical Association*.

[10] Chasman D. I., Giulianini F., MacFadyen J., Barratt B. J., Nyberg F., Ridker P. M. (2012). Genetic determinants of statin-induced low-density lipoprotein cholesterol reduction: the Justification for the Use of Statins in Prevention: an Intervention Trial Evaluating Rosuvastatin (JUPITER) trial. *Circulation: Cardiovascular Genetics*.

[11] Pedro-Botet J., Schaefer E. J., Bakker-Arkema R. G. (2001). Apolipoprotein E genotype affects plasma lipid response to atorvastatin in a gender specific manner. *Atherosclerosis*.

[12] O'Neill F. H., Patel D. D., Knight B. L. (2001). Determinants of variable response to statin treatment in patients with refractory familial hypercholesterolemia. *Arteriosclerosis, Thrombosis, and Vascular Biology*.

[13] Swirski F. K., Libby P., Aikawa E. (2007). Ly-6Chi monocytes dominate hypercholesterolemia-associated monocytosis and give rise to macrophages in atheromata. *Journal of Clinical Investigation*.

[14] Robbins C. S., Chudnovskiy A., Rauch P. J. (2012). Extramedullary hematopoiesis generates Ly-6C(high) monocytes that infiltrate atherosclerotic lesions. *Circulation*.

[15] Murphy A. J., Akhtari M., Tolani S. (2011). ApoE regulates hematopoietic stem cell proliferation, monocytosis, and monocyte accumulation in atherosclerotic lesions in mice. *Journal of Clinical Investigation*.

[16] Soehnlein O., Swirski F. K. (2013). Hypercholesterolemia links hematopoiesis with atherosclerosis. *TrendsEndocrinology & Metabolism*.

[17] van der Wal A. C., Becker A. E. (1999). Atherosclerotic plaque rupture–pathologic basis of plaque stability and instability. *Cardiovascular Research*.

[18] Hirschfield G. M., Pepys M. B. (2003). C-reactive protein and cardiovascular disease: new insights from an old molecule. *QJM: An International Journal of Medicine*.

[19] Lau D. C., Dhillon B., Yan H., Szmitko P. E., Verma S. (2005). Adipokines: molecular links between obesity and atheroslcerosis. *American Journal of Physiology-Heart and Circulatory Physiology*.

[20] Bernot D., Benoliel A., Peiretti M. (2003). Effect of atorvastatin on adhesive phenotype of human endothelial cells activated by tumor necrosis factor alpha. *Journal of Cardiovascular Pharmacology*.

[21] Romano M., Diomede L., Sironi M. (2000). Inhibition of monocyte chemotactic protein-1 synthesis by statins. *Laboratory Investigation*.

[22] Laufs U., Liao J. K. (2003). Isoprenoid metabolism and the pleiotropic effects of statins. *Current Atherosclerosis Reports*.

[23] Ridker P. M., Rifai N., Clearfield M. (2001). Measurement of C-reactive protein for the targeting of statin therapy in the primary prevention of acute coronary events. *New England Journal of Medicine*.

[24] Ridker P. M., Cannon C. P., Morrow D. (2005). C-reactive protein levels and outcomes after statin therapy. *New England Journal of Medicine*.

[25] Knopp R. H. (1999). Drug treatment of lipid disorders. *New England Journal of Medicine*.

[26] Camnitz W., Burdick M. D., Strieter R. M., Mehrad B., Keeley E. C. (2012). Dose-dependent effect of statin therapy on circulating CXCL12 levels in patients with hyperlipidemia. *Clinical and Translational Medicine*.

[27] van der Meij E., Koning G. G., Vriens P. W. (2013). A clinical evaluation of statin pleiotropy: statins selectively and dose-dependently reduce vascular inflammation. *PLoS One*.

[28] Santosa S., Varady K., AbuMweis A. S., Jones P. J. (2007). Physiological and therapeutic factors affecting cholesterol metabolism: does a reciprocal relationship between cholesterol absorption and synthesis really exist?. *Life Sciences*.

[29] Mangat R., Warnakula S., Wang Y. (2010). Model of intestinal chylomicron over-production and ezetimibe treatment: impact on the retention of cholesterol in arterial vessels. *Atherosclerosis Supplements*.

[30] Vine D. F., Takechi R., Russell J. C., Proctor S. D. (2007). Impaired postprandial apolipoprotein-B48 metabolism in the obese, insulin-resistant JCR:LA-cp rat: increased atherogenicity for the metabolic syndrome. *Atherosclerosis*.

[31] Proctor S. D., Vine D. F., Mamo J. C. (2002). Arterial retention of apolipoprotein B(48)- and B(100)-containing lipoproteins in atherogenesis. *Current Opinion in Lipidology*.

[32] Proctor S. D., Vine D. F., Mamo J. C. (2004). Arterial permeability and efflux of apolipoprotein B-containing lipoproteins assessed by in situ perfusion and three-dimensional quantitative confocal microscopy. *Arteriosclerosis, Thrombosis, and Vascular Biology*.

[33] Silbernagel G., Fauler G., Renner W. (2009). The relationships of cholesterol metabolism and plasma plant sterols with the severity of coronary artery disease. *Journal of Lipid Research*.

[34] Matthan N. R., Pencina M., LaRocque J. M. (2009). Alterations in cholesterol absorption/synthesis markers characterize Framingham offspring study participants with CHD. *Journal of Lipid Research*.

[35] Weingartner O., Weingartner N., Scheller B. (2009). Alterations in cholesterol homeostasis are associated with coronary heart disease in patients with aortic stenosis. *Coronary Artery Disease*.

[36] Weingartner O., Lutjohann D., Bohm M., Laufs U. (2010). Relationship between cholesterol synthesis and intestinal absorption is associated with cardiovascular risk. *Atherosclerosis*.

[37] Ference B. A., Majeed F., Penumetcha R., Flack J. M., Brook R. D. (2015). Effect of naturally random allocation to lower low-density lipoprotein cholesterol on the risk of coronary heart disease mediated by polymorphisms in NPC1L1, HMGCR, or both: a 2 × 2 factorial Mendelian randomization study. *Journal of American College of Cardiology*.

[38] Buchwald H., Varco R. L., Matts J. P. (1990). Effect of partial ileal bypass surgery on mortality and morbidity from coronary heart disease in patients with hypercholesterolemia. Report of the Program on the Surgical Control of the Hyperlipidemias (POSCH). *New England Journal of Medicine*.

[39] Cannon C. P., Blazing M. A., Braunwald E. (2015). Ezetimibe plus a statin after acute coronary syndromes. *New England Journal of Medicine*.

[40] Chan J. C., Kong A. P., Bao W., Fayyad R., Laskey R. (2016). Safety of atorvastatin in Asian patients within clinical trials. *Cardiovascular Therapeutics*.

[41] Liao J. K. (2007). Safety and efficacy of statins in Asians. *The American Journal of Cardiology*.

[42] Park M. W., Park G. M., Han S. (2018). Moderate-intensity versus high-intensity statin therapy in Korean patients with angina undergoing percutaneous coronary intervention with drug-eluting stents: a propensity-score matching analysis. *PLoS One*.

