# Supplementary material for: Roles of Achieved Levels of Low-Density Lipoprotein Cholesterol and High-Sensitivity C-Reactive Protein on Cardiovascular Outcome in Statin Therapy
Source: Cardiovasc Ther. 2019 Nov 21;2019:3824823. doi: 10.1155/2019/3824823 (PMC6906885; doi:10.1155/2019/3824823)
Supplement: Supplementary Materials — Table S1: baseline characteristics of co-medications usage according to quartiles of achieved LDL-C level. Table S2: baseline characteristics of co-medications usage according to quartiles of followed-up hsCRP level. Figure S1: study flow chart. [file 3824823.f1.docx]

**TABLE S1.** Baseline characteristics of co-medications usage according to quartiles of achieved LDL-C level.

|  | **LDL-C** | | | |  |
| --- | --- | --- | --- | --- | --- |
| **Variables** | **Quartile 1  (<1.74 mmol/L)** | **Quartile 2  (1.74-2.15 mmol/L)** | **Quartile 3  (2.15-2.67 mmol/L)** | **Quartile 4  (≥2.67 mmol/L)** | **p Value** |
| Aspirin, n (%) | 635 (51.8%) | 540 (45.2%) | 525 (44.2%) | 477 (39.9%) | < 0.001 |
| Clopidogrel, n (%) | 163 (13.3%) | 165 (13.8%) | 121 (10.2%) | 96 (8.0%) | < 0.001 |
| Cilostazol, n (%) | 119 (9.7%) | 72 (6.0%) | 73 (6.1%) | 65 (5.4%) | < 0.001 |
| Warfarin, n (%) | 175 (14.3%) | 116 (9.7%) | 110 (9.3%) | 102 (8.5%) | < 0.001 |
| BB, n (%) | 224 (18.3%) | 291 (24.3%) | 310 (26.1%) | 283 (23.7%) | 0.001 |
| Diuretics, n (%) | 338 (27.6%) | 337 (28.2%) | 369 (31.1%) | 335 (28.1%) | 0.450 |
| ARB, n (%) | 598 (48.8%) | 554 (46.3%) | 529 (44.6%) | 485 (40.6%) | < 0.001 |
| ACEi, n (%) | 95 (7.7%) | 98 (8.2%) | 118 (9.9%) | 89 (7.5%) | 0.802 |
| CCB, n (%) | 539 (44%) | 556 (46.5%) | 553 (46.6%) | 475 (39.8%) | 0.054 |
| DHP, n (%) | 409 (33.4%) | 424 (35.5%) | 427 (36%) | 386 (32.3%) | 0.684 |
| non-DHP, n (%) | 173 (14.1%) | 193 (16.1%) | 174 (14.7%) | 137 (11.5%) | 0.038 |
| Nitrate, n (%) | 182 (14.8%) | 174 (14.5%) | 166 (14%) | 137 (11.5%) | 0.016 |
| Trimetazidine, n (%) | 145 (11.8%) | 121 (10.1%) | 120 (10.1%) | 111 (9.3%) | 0.052 |
| Nicorandil, n (%) | 106 (8.6%) | 102 (8.5%) | 97 (8.2%) | 63 (5.3%) | 0.002 |
| Molsidomine, n (%) | 19 (1.5%) | 17 (1.4%) | 12 (1.0%) | 14 (1.2%) | 0.288 |

LDL-C, low-density lipoprotein cholesterol; BB, beta blocker; ARB, angiotensin II receptor blocker; ACEi, angiotensin converting enzyme inhibitor; CCB, calcium channel blocker; DHP, dihydropyridine; non-DHP, non-dihydropyridine

**TABLE S2.** Baseline characteristics of co-medications usage according to quartiles of followed-up hsCRP level.

|  | **hsCRP** | | | |  |
| --- | --- | --- | --- | --- | --- |
| **Variables** | **Quartile 1  (<35.1 μmol/L)** | **Quartile 2  (35.1-69.4 μmol/L)** | **Quartile 3  (69.4-141.8 μmol/L)** | **Quartile 4  (>141.8 μmol/dL)** | **p Value** |
| Aspirin, n (%) | 538 (44.0%) | 535 (45.0%) | 546 (45.7%) | 558 (46.7%) | 0.160 |
| Clopidogrel, n (%) | 145 (11.8%) | 117 (9.8%) | 114 (9.5%) | 169 (14.2%) | 0.112 |
| Cilostazol, n (%) | 79 (6.5%) | 74 (6.2%) | 95 (7.9%) | 81 (6.8%) | 0.405 |
| Warfarin, n (%) | 90 (7.4%) | 101 (8.5%) | 129 (10.8%) | 183 (15.3%) | < 0.001 |
| BB, n (%) | 317 (25.9%) | 258 (21.7%) | 227 (19%) | 306 (25.6%) | 0.493 |
| Diuretics, n (%) | 329 (26.9%) | 318 (26.7%) | 322 (26.9%) | 410 (34.3%) | < 0.001 |
| ARB, n (%) | 531 (43.4%) | 527 (44.3%) | 517 (43.2%) | 591 (49.5%) | 0.007 |
| ACEi, n (%) | 111 (9.1%) | 98 (8.2%) | 89 (7.4%) | 102 (8.5%) | 0.498 |
| CCB, n (%) | 599 (48.9%) | 517 (43.5%) | 483 (40.4%) | 524 (43.9%) | 0.004 |
| DHP, n (%) | 449 (36.7%) | 396 (33.3%) | 383 (32%) | 418 (35%) | 0.293 |
| non-DHP, n (%) | 222 (18.1%) | 162 (13.6%) | 142 (11.9%) | 151 (12.6%) | < 0.001 |
| Nitrate, n (%) | 197 (16.1%) | 159 (13.4%) | 132 (11%) | 171 (14.3%) | 0.080 |
| Trimetazidine, n (%) | 162 (13.2%) | 120 (10.1%) | 99 (8.3%) | 116 (9.7%) | 0.001 |
| Nicorandil, n (%) | 123 (10.0%) | 94 (7.9%) | 66 (5.5%) | 85 (7.1%) | 0.001 |
| Molsidomine, n (%) | 15 (1.2%) | 18 (1.5%) | 14 (1.2%) | 15 (1.3%) | 0.867 |

hsCRP, high-sensitivity c-reactive protein low-density lipoprotein cholesterol; BB, beta blocker; ARB, angiotensin II receptor blocker; ACEi, angiotensin converting enzyme inhibitor; CCB, calcium channel blocker; DHP, dihydropyridine; non-DHP, non-dihydropyridine

**Figure S1.** Study flow chart


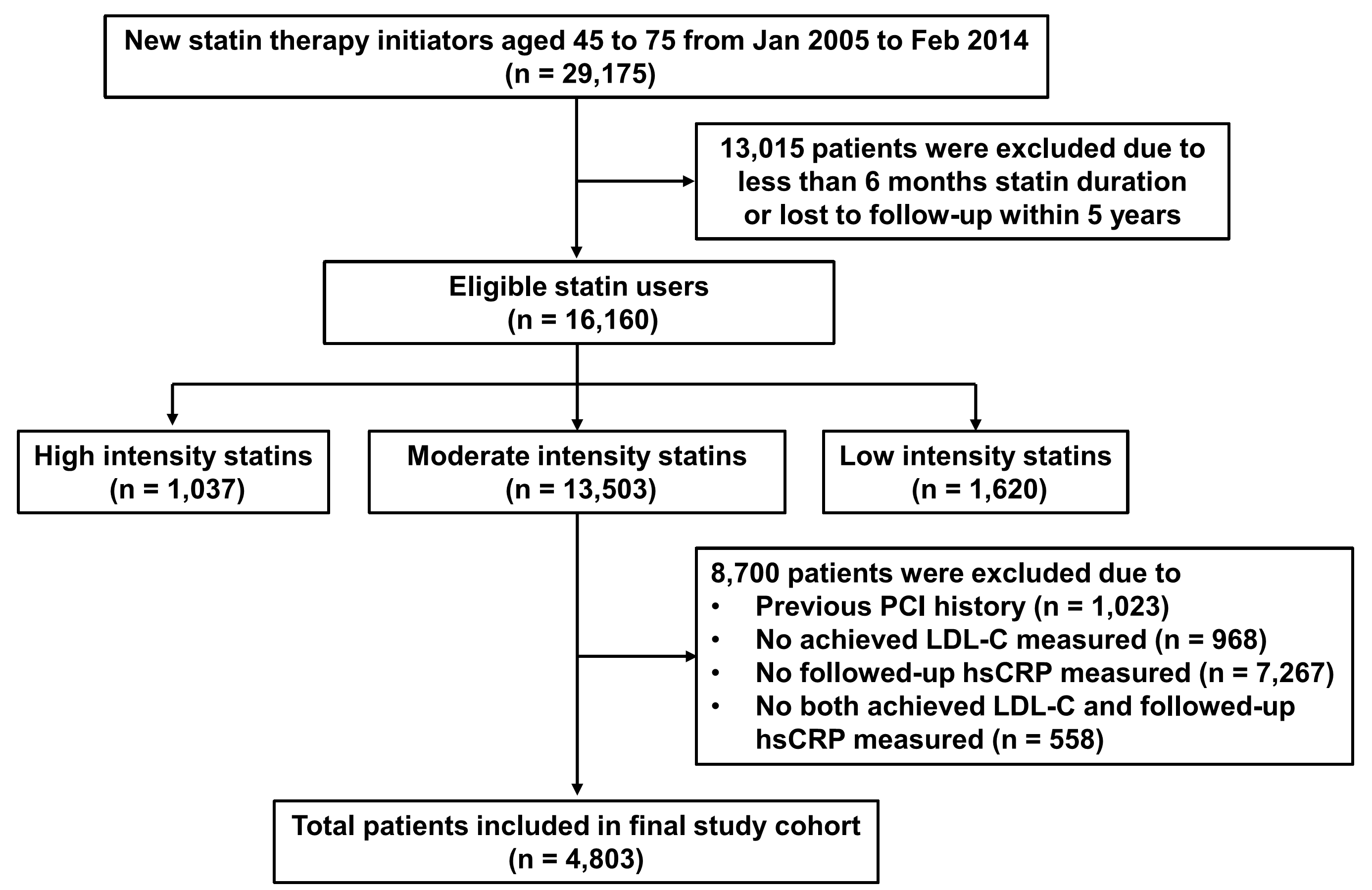


PCI, percutaneous coronary intervention; LDL-C, low-density lipoprotein cholesterol; hsCRP, high-sensitivity c-reactive protein
